# Delivering digital cognitive behavioral therapy for insomnia at scale: does using a wearable device to estimate sleep influence therapy?

**DOI:** 10.1038/s41746-017-0010-4

**Published:** 2018-02-19

**Authors:** Annemarie I. Luik, Pedro Farias Machado, Colin A. Espie

**Affiliations:** 1 Sleep and Circadian Neuroscience Institute, Nuffield Department of Clinical Neurosciences, University of Oxford, Oxford, United Kingdom; 2 Big Health Ltd., London, United Kingdom

**Keywords:** Therapeutics, Health services

## Abstract

Contemporary developments, such as digital Cognitive Behavioral Therapy (CBT) and wearable devices estimating sleep, could support the implementation of CBT for insomnia at a large scale. We assessed what characterizes those users who connected a wearable device to the program to estimate sleep diary variables, and whether connecting a wearable device affected insomnia symptom improvement, related well-being, and program interaction. In total, 3551 users (63% female, mean age 44.50 ± 14.78 years) of a dCBT program who completed a post-therapy survey, including 378 users (10.6%) who used a device, were selected. Within-subject, pre-therapy to post-therapy, the Sleep Condition Indicator (SCI, 7 Items) was used to assess insomnia. Two-item measures (depression, anxiety) and single item measures (perceived stress, life satisfaction, work productivity) of well-being were analyzed, in addition to program interaction. For all participants, insomnia symptoms significantly improved following dCBT (t(3504) = 83.33, *p* < 0.001; Cohen’s *d* = 1.45), as did depression and anxiety symptoms, perceived stress, life satisfaction and work productivity. Those who did not connect a device reported better sleep and less affected work productivity (all *p* < .001) than those who did connect a device at baseline and post-treatment; nevertheless treatment effects were largely similar for the two groups. Those who connected a device interacted more with additional program components. In conclusion, improvements in insomnia after completing dCBT are similar in persons choosing to wear a wearable device to estimate sleep and persons completing a subjective sleep diary. Potentially, use of wearable devices can facilitate treatment for those who struggle to complete daily diaries.

## Digital therapy: Wearable sleep trackers do not change effectiveness of digital insomnia intervention

People who track their sleep with wearable devices and self-recorded diaries get the same benefit from an online sleep-improvement program. Annemarie Luik from the University of Oxford, UK, and colleagues compared the effectiveness of a digital insomnia intervention called Sleepio among 378 people who wore FitBit or Jawbone devices to estimate their sleep and more than 3,000 others who relied on more subjective sleep diaries. All participants received six weekly sessions of cognitive behavioral therapy with a virtual personal therapist. Those who used a wearable device interacted more with the program and perceived their insomnia as worse. However, both groups, regardless of how they kept tabs on their sleep, had similar improvements in insomnia symptoms and associated wellbeing. The authors conclude that digital CBT is equally effective with or without using a wearable device to estimate sleep.

## Introduction

Cognitive Behavioral Therapy (CBT) received renewed attention when it was recommended recently as the treatment of choice for insomnia by the American College of Physicians.^1^ The publication of these guidelines, however, also attracted comments in relation to a number of substantial implementation challenges, such as the scalability of the treatment.^2^ Part of the solution to the challenge of disseminating CBT more widely could be the introduction of digital CBT (dCBT)^3^ where CBT is provided by digital means, such as a mobile application or computer. A number of such programs has been developed in the last decade (for example^4–9^), and a recent meta-analysis concluded that the effects of dCBT are in the range of the effect sizes for face-to-face CBT.^10^ Direct comparisons suggest that face-to-face CBT is superior to dCBT,^11^ however, there was no difference between group CBT and dCBT in a small trial.^9^

In addition to the emergence of digital therapy, the ubiquitous nature of commercially available wearable devices brings fresh challenges and opportunities. Evidence on how well these wearables estimate sleep in healthy persons is mixed.^12–14^ Furthermore, the validity of wearable devices in insomnia is largely unknown; although certain accelerometers may estimate sleep quite reliably,^15–17^ suggesting they might be a useful tool for treatment. For decades, sleep has been assessed with a daily paper-and-pen sleep diary (e.g.^18,19^) and the consensus diary^20^ is now recommended as a valid tool in those suffering from insomnia.^21^ This “traditional” approach, however, can be burdensome for many patients and can result in levels of missing or, at best, estimated data.^22^ Perhaps, wearable devices offer a user-friendly way to track sleep as part of insomnia treatment.^23^ Moreover, because some dCBT programs, such as the program used in this evaluation,^5^ can integrate wearable data, there may be potential to tailor the dCBT treatment to the patient’s sleep. However, it is not known if or how the use of a wearable estimating sleep during dCBT affects therapy outcomes. As insomnia is based on subjective criteria it might be that the self-assessment of sleep in a sleep diary is integral to the treatment, and that wearing a device potentially takes away this self-assessing component. On the other hand, measuring sleep data objectively might force reflection on the objective components of sleep and not only subjectively experienced sleep.

First, we assessed the effectiveness of a dCBT for insomnia in a real-world sample of persons who completed *Sleepio™*, a program which has been found to be effective in formal RCTs in improving insomnia.^5,24–27^ We expected to confirm that dCBT can successfully improve insomnia and related well-being constructs in an evaluation of an ongoing service, similar to the previous evidence from RCTs. However, our main research aims were to (1) assess what characterizes those users who connected a wearable device to the program to estimate sleep diary variables, (2) assess whether connecting a wearable device affected insomnia symptom improvement and improvement on related well-being factors, and (3) assess whether connecting a wearable device affected the interaction with the program compared to those who did not connect a wearable device. In an effort to identify the effect of using a device in the optimized treatment situation, we only focus upon those who completed treatment, to ensure that participants had actually utilized their sleep diary data on a regular basis, including throughout the core component of CBT for insomnia, sleep restriction, which extends from session three through to the final session six. The essential difference of interest, therefore, was between those whose diary was self-completed throughout (the default state of the Sleepio program) and those who connected a device to automatically fill the diary throughout. Reporting upon completers also provides us with the best comparison between these sub-groups because the program routinely incorporates a post-test after the sixth session. We expected improvement on insomnia symptoms and well-being irrespective of connecting a device. As there is limited to no evidence on the use of wearable devices in behavior change programs, we did not specify a direction of possible differences in treatment effect between those connecting a device to the dCBT program to estimate diaries and subjectively completing diaries.

## Results

### Descriptives

Of a total of 3551 dCBT completers in this report, 378 users connected a wearable device to the program (10.6%). A similar number of people connected a Fitbit® (*N* = 183) or a Jawbone UP*™* (*n* = 195). The full sample comprised more women (63%), had a mean age of 44.5 years, 70% of users were employed full-time and 6.8% of the sample reported to be a shift worker. About 35% perceived themselves as overweight, 52% used alcohol for more than once a week and 73% reported the use of caffeine at least once a day. Only 11% reported as smokers while a large majority exercised for 30 min at least once per week. Persons who connected a device to the program did not differ from those who did not connect a device (see Table 1), except for the amount of exercising and use of prescribed sleep medication. Those who connected a device were more likely to exercise more *χ*^2^ (4) = 11.676, *p* = 0.020) and use medication than those who did not connect a device (*χ*^2^ (1) = 4.47, *p* = 0.034).

**Table 1:** Demographics and baseline lifestyle descriptives for the full sample, users who have not connected a device and who have connected a device

	All users	No device users	Device users	No device vs. device	
	(*n* = 3551)	(*n* = 3173)	(*n* = 378)		
	*N* (%)/mean ± SD	*N*(%)/mean ± SD	*N*(%)/mean ± SD	Test-statistic	*p*
Female (yes)	2233 (62.9%)	2010 (63.3%)	223 (59.0%)	*χ*^2^(1) = 2.835	0.092
Age (years)	44.50 ± 14.78	44.47 ± 14.93	44.71 ± 13.56	*F*(1) = 0.089	0.766
Employed (yes)	2496 (70.3%)	2221 (70.0%)	275 (72.8%)	*χ*^2^(1) = 2.314	0.128
Shift worker (yes)	243 (6.8%)	219 (6.9%)	24 (6.3%)	*χ*^2^(1) = 0.002	0.963
Self-rated overweight (yes)	1245 (35.1%)	1104 (34.8%)	141 (37.3%)	*χ*^2^(1) = 1.612	0.204
Alcohol use^a^				*χ*^2^(4) = 5.001	0.287
Never	616 (17.4%)	549 (17.3%)	67 (17.7%)		
Less than once a week	1085 (30.6%)	985 (31.1%)	100 (26.5%)		
Once a week	616 (17.4%)	553 (17.4%)	63 (16.7%)		
2–3 times a week	799 (22.5%)	705 (22.2%))	94 (24.9%)		
>4 times a week	434 (12.2%)	380 (12.0%)	54 (14.3%)		
Caffeine use^a^				*χ*^2^(4) = 5.345	0.254
Never	379 (10.7%	343 (10.8%)	36 (9.5%)		
Less than once a day	585 (16.5%)	533 (16.8%)	52 (13.8%)		
Once a day	1056 (29.7%)	941 (29.7%)	115 (30.4%)		
2–3 times a day	1200 (33.8%)	1056 (33.3%)	144 (38.1%)		
>4 times a day	330 (9.3%)	299 (9.4%)	31 (8.2%)		
Tobacco use^a^				*χ*^2^(4) = 2.433	0.657
Never smoked	3162 (89.1%)	2831 (89.2%)	331 (87.6%)		
Rarely	239 (6.7%)	213 (6.7%)	26 (6.9%)		
1–10 times a day	107 (3.0%)	92 (2.9%)	15 (4.0%)		
10–20 times a day	32 (0.9%)	28 (0.9%)	4 (1.1%)		
>21 times a day	10 (0.3%)	8 (0.3%)	2 (0.5%)		
Exercising > 30 min^b^				χ^2^(4) = 11.676	0.020
Never	178 (5.2%)	166 (5.4%)	12 (3.3%)		
Less than once a week	452 (13.1%)	407 (13.2%)	45 (12.2%)		
Once a week	591 (17.1%)	530 (17.2%)	61 (16.6%)		
2–3 times a week	1227 (35.5%)	1110 (36.0%)	117 (31.8%)		
>4 times a week	1007 (29.1%)	874 (28.3%)	133 (36.1%)		
Sleep medication (yes)	813 (22.9%)	712 (22.6%)	101 (27.5%)	χ^2^(1) = 4.472	0.034

*SD* Standard deviation

^a^ Missing *n* = 1

^b^ Missing *n* = 46

### Treatment outcomes

First, we assessed the effects of dCBT in the full sample (see Table 2), the post treatment test was completed with a median of 42 days (InterQuartile Range (IQR): 37–54) after the start of session 1. The median of completed diaries during this period was 41 (IQR: 36–49). Overall sleep quality on the SCI significantly improved after dCBT for insomnia (*t*(3504) = 83.33, *p* < 0.001; Cohen’s *d* = 1.45 [95% CI 1.41–1.50]). Results stratified for the use of prescribed sleep medication can be found in supplementary table 1, suggesting that the change in SCI is larger for those who use medication than those who do not.

**Table 2:** Sleep, depression, anxiety, perceived stress, overall health, life satisfaction and productivity at baseline and post-treatment for all users (*n* = 3551)

	Baseline		Post-treatment		Test-statistic	*p*
	*N*	Mean ± SD/ median (IQR)	*N*	Mean ± SD/ median (IQR)		
Sleep (SCI-7)	3533	4.25 ± 1.94	3522	7.15 ± 2.04	*t*(3504) = 83.33^b^	<0.001
Depression (PHQ-2)	3495	2 (0–3)	3524	1 (0–2)	*Z* = −26.81^c^	<0.001
Anxiety (GAD-2)	3495	2 (1–3)	3524	1 (0–2)	*Z* = −29.51^c^	<0.001
Perceived stress	3444	2 (1–2)	3524	1 (0–2)	*Z* = −28.69^c^	<0.001
Life satisfaction	3466	7 (5–8)	3523	7 (6–8)	*Z* = −19.16^d^	<0.001
Work productivity	2460^a^	2 (1–4)	3444	1 (0–3)	*Z* = −25.60^c^	<0.001

*SD* standard deviation, *IQR* interquartile range, *SCI*-7 sleep condition indicator 7 items, *PHQ-2* Patient Health Questionnaire 2 items, *GAD-2* generalize anxiety disorder 2 items

^a^ Baseline work productivity is only assessed in those who were employed, *n* = 2496

^b^ Paired T-test

^c^ Wilcoxon signed rank test, Z-value based on negative ranks

^d^ Wilcoxon signed rank test, Z-value based on positive ranks

Significant reductions in depressive symptoms (*Z* = −26.81, *p* < 0.001), symptoms of anxiety (*Z* = −29.51, *p* < 0.001), perceived stress (*Z* = −28.69, *p* < 0.001), life dissatisfaction (*Z* = −19.16, *p* < 0.001) and less poor work productivity (*Z* = −25.42, *p* < 0.001) were observed following dCBT (see Table 2). These results remained similar after the exclusion of shift workers.

The results in Table 3 demonstrate that users with no connected device had a significantly better sleep and less sleep affected work productivity than device users both at baseline (respectively F(1,3532) = 12.94, *p* < 0.001 and *U* = 233095, *p* < 0.001) and at post-therapy (respectively, F(1,3521) = 11.84, *p* < 0.001 and *U* = 489106, *p* < 0.001). In addition, those who did not connect a device had less depressive symptoms at post-therapy than those who connected a device (*U* = 553788, *p* < 0.001). Similar to the entire user group, therapy effects were significant for all variables for both those who connected a device and those who did not connect a device (all *p* ≤ 0.001, see supplementary table 2). Further analyses of change scores, i.e. post-treatment scores minus baseline scores, demonstrated that the therapy effect did not differ for change in insomnia between those who connected a device and those who did not. Of the well-being outcomes only the change in work productivity differed between those who connected a device and those who did not, where the decrease was slightly smaller in those who connected a device (*U* = 256138, *p* = 0.009). Stratification for medication demonstrated that the difference in work productivity between those who used a device and those who did not was only significant for those who did not use medication (*U* = 144305, *p* = 0.008).

**Table 3:** Sleep, depression, anxiety, perceived stress, overall health, life satisfaction and productivity at baseline and post-treatment for users who did not connect a device (*n* = 3173) vs. user who connected a device (*n* = 378)

	Baseline						Post-treatment					
	No device		Device				No device		Device			
	Mean ± SD/ median (IQR)	Mean rank	Mean ± SD/ median (IQR)	Mean rank	Test-statistic	*p*	Mean ± SD/ median (IQR)	Mean rank	Mean ± SD/ median (IQR)	Mean rank	Test-statistic	*p*
Sleep (SCI-7)	4.29 ± 1.97	–	3.91 ± 1.60	–	F(1,3532) = 12.94^a^	<0.001	7.19 ± 2.03	–	6.80 ± 2.08	–	F(1,3521) = 11.84^a^	<0.001
Depression (PHQ-2)	2 (0–3)	1743	2 (0–3)	1792	*U* = 545748^b^	0.372	1 (0–2)	1751	1 (0–2)	1857	*U* = 553788^b^	0.042
Anxiety (GAD-2)	2 (1–3)	1748	2 (1–3)	1746	*U* = 560727^b^	0.964	1 (0–2)	1761	1 (0–2)	1774	*U* = 584480^b^	0.796
Perceived stress (PSS)	2 (1–2)	1714	2 (1–3)	1797	*U* = 516589^b^	0.126	1 (0–2)	1755	1 (0–2)	1825	*U* = 565763^b^	0.187
Life satisfaction	7 (5–8)	1727	7 (6–8)	1795	*U* = 522368^b^	0.216	7 (6–8)	1765	7 (6–8)	1737	*U* = 579655^b^	0.216
Work productivity	2 (1–4)	1205	3 (2–4)	1442	*U* = 233095^b^	<0.001	1 (0–3)	1700	2 (0–3)	1915	*U* = 489106^b^	<0.001

*SD* standard deviation, *IQR* interquartile range, *SCI-7* Sleep Condition Indicator 7 items, *PHQ-2* Patient Health Questionnaire 2 items,* GAD-2* generalize anxiety disorder 2 items

^a^ ANOVA

^b^ Mann–Whitney U test

### Program interaction

Interaction with the online program was evaluated by assessing several metrics collected within the program. Users with a device were more likely to view the library (difference: 9.4%, *χ*^2^(1) = 16.61, *p* < 0.001) and more likely to post in the community (difference 5.5%, *χ*^2^(1) = 9.41, *p* = 0.002). Although we did not see a difference in the percentage of people who viewed the community (*χ*^2^(1) < 0.001, *p* = 0.983), we did see that people with a device were viewing the community more often (median difference: 3, *U* = 538614, *p* = 0.001). No difference in number of diaries completed was found.

## Discussion

This evaluation suggests that, within a sample of persons who complete dCBT, persons who choose to wear a device to estimate their sleep reported more severe insomnia complaints, more use of sleep medication and more affected work productivity than persons who manually complete sleep diaries users of devices. They did not differ majorly with regards to demographics and lifestyle, although users who connected a device were more likely to exercise more often. Both groups had similar improvements in insomnia and associated well-being, although those who connected a device tended to interact with the program more. In addition, the results lend support to the validity of controlled trial data from numerous studies (typical *n* = 100–200) suggesting that dCBT improves insomnia symptoms in a large sample of treatment completers.

In our sample of dCBT completers, 11% of users connected a wearable device to the program, which is at the lower end of published estimates of how many people own a device.^28,29^ However, usage of wearable devices is shown to drop by one-third after 6 months of buying a wearable device, and to half after 18 months of buying.^30^ In addition, not everyone might want to connect their device to the program. Although possible, it seems unlikely that people have specifically bought a wearable device for the dCBT program. This could be due to many reasons such as price, comfort, esthetics or other factors, not necessarily related to the preference of completing diaries online or via a device within a dCBT program. Users that connected a device had poorer sleep at the start of the program and, possibly associated with this, a higher use of sleep medication and reports of poorer work-productivity due to sleep. Perhaps observing your device-generated data influences your perspective on your sleep and the need to pursue a sleep intervention. Alternatively, those with more severe sleep problems or more severe daytime effects because of sleep problems might also be more likely to use a device to assess their sleep. Users that connected a device within this sample did not differ on any other measured demographic, lifestyle or well-being factors, except the amount of exercise.

Importantly, connecting a device to the program did not influence the effectiveness of dCBT substantially, only a minor difference in therapy effect on work productivity was seen. This might be surprising considering the validity of commercially available wearable devices is largely unknown. We cannot however interpret the lack of difference in treatment outcomes as evidence for the validity of commercially available wearable devices, as the current study is not set up to validate measures. However, when measurement errors are consistent at each time point, they will have a limited impact on change scores, as the same error will be reflected at each measurement. We therefore suggest that using a device to estimate sleep during dCBT is a matter largely of preference, and that if it is the preferred method of the user, connecting a device to dCBT will not affect treatment outcomes. It is possible that the device group may have been less likely to complete online daily sleep diaries manually, and so devices may reduce user burden. As we did not use randomization is this evaluation, we cannot assess the effect of assigning a device to estimate sleep diaries independently of user preference. In addition, integration of devices to face-to-face treatment might differ from integration to dCBT. Pilot data however suggests that when the use of a device is assigned at random within a combined face-to-face and digital treatment there are equally no differences in treatment outcomes.^17^ Therefore, we suggest that devices can be integrated to dCBT, and possibly also face-to-face treatment, if the patient prefers to use a wearable device to estimate their sleep without affecting treatment effectiveness. Possibly, clinicians could also suggest wearable devices as a tool for those who specifically struggle to keep pen-and-paper sleep diaries.

In addition, we found significant differences in interaction with the program between those who connected a device to the program and those who do not. Using a device was associated with greater use of program components such as viewing the library and active posting on the community. Perhaps those connecting a device were more comfortable with technology, or alternatively, were more motivated to use the full range of tools available due to more severe sleep problems.

Within this evaluation, we specifically concentrated on determining whether using a wearable to estimate sleep diary data or manually completing sleep diaries within a dCBT program would change treatment outcomes. To answer this question, we have chosen to assess those who utilized the sleep diary for a longer time period, allowing us to assess the impact of automated diary completion on the outcomes of the full treatment, including sleep restriction which is dependent on daily sleep information. CBT however suffers from ineffectiveness because of two separate user journeys. First, persons who complete treatment but do not show improvement and second, persons who do not complete treatment and therefore do not improve. While we have focused on the first category of user journeys, the second category of user journeys is naturally also important to study in future research, for example by assessing whether persons are quitting therapy early because they do not respond to therapy or whether they are quitting early because they respond early and do not need any further treatment. dCBT could provide the opportunity to study these user journeys at a large scale.

Evaluating data at this large scale comes with advantages such as a higher generalizability and increased statistical power. Importantly, we also avoid measurement bias by using outcomes that are measured by questionnaire in both groups, and not comparing sleep diary measures, since these outcomes would be estimated by devices for the device users and self-rated for those who do not use a device. However, this evaluation also has several limitations. First, to be able to ensure our research question we assessed a sample of users that had completed a post-therapy survey, making it likely that the cohort comprised motivated individuals. This can have inflated the overall treatment effects. In line with our research question, we evaluated existing data on a large scale and hence were not able to have a control group such as in a RCT. Current results align however with those suggested from previous controlled trials. Lastly, users who connected a device to the program might have differed on baseline characteristics that we did not measure, and post-therapy outcomes other than the ones we evaluated may have revealed group differences in treatment outcomes. Use of devices within the program was self-chosen, a future RCT in which the use of wearable devices is randomized might give us more insight in non-self-selected used of wearable devices and treatment effects more extensively.

In conclusion, this evaluation confirms that integration of wearable device data may offer new opportunities for dCBT, and although validation of device generated sleep estimates remains elusive, participants in this sample of dCBT completers achieved outcomes comparable to those who did not use wearable devices. In addition, we found some evidence that they were more likely to utilize the full range of options available within the dCBT program.

## Methods

### Participants

This is an evaluation of data collected within an online sleep improvement program (*Sleepio™*, Big Health Ltd., London, UK). All users consent to the anonymized use of their data when they access the program (www.sleepio.com/privacy and www.sleepio.com/terms). In addition, the program is fully HIPAA and HITRUST compliant. As this manuscript presents an evaluation of an ongoing service no approval of a research ethics committee was obtained. Users can be self-referred, referred by clinicians or have access through an employer’s wellbeing offering. For the present report, we selected a cohort of users who completed the program and a recently introduced post-intervention assessment, in an effort to identify the effect of using a device on full treatment outcomes.

### Intervention

*Sleepio™* is an online, fully automated, dCBT program for insomnia.^5^ Users receive six weekly sessions from an animated personal therapist in which core behavioral techniques, such as sleep restriction (i.e. reducing the sleep window to enhance sleep consolidation) and stimulus control (i.e., getting out of bed after 15–20 min of being awake), and cognitive techniques, such as thought re-structuring (i.e. targeting unrealistic thoughts about sleep and the effects of sleeping less) and paradoxical intention (i.e. trying to stay awake instead of trying to sleep), are systematically introduced. Sessions are tailored based on user characteristics and a so-called daily sleep diary. The diary is based on the Consensus Sleep Diary^20^ and asks the user several questions about the quanity and quality of their sleep each day, for example ‘How long did it take you to fall asleep? and ‘How would you rate the quality of your sleep?’. These questions are used to inform one of the core components of CBT for insomnia which is called sleep restriction. Sleep restriction is a technique in which the sleep window (time in bed) is reduced to enhance sleep consolidation. The technique is introduced in session three and the initial suggested sleep window is calculated from the sleep diary data of the previous weeks. If the sleep diary data indicate a sleep efficiency (total sleep time/time in bed*100) of 90% or higher in the following sessions, the animated therapist advises that 15 min is added to the sleep window until the optimal sleep window is established. In addition to the six weekly sessions, there is access to additional components such as an online community of users and a library with background information (for a more detailed description please refer to^5^). The intervention has been evaluated in a randomized placebo controlled clinical trial^5,31^ Results indicate that the program can be used successfully to improve sleep quality and sleep as estimated by self-rated online sleep diaries. More recent trials have confirmed such findings, and suggest that the program is also associated with more generalized health and workplace benefits.^24–27^

The daily collection of sleep data relies on users adding daily online sleep diaries to their ‘case file’. By default, users must input all sleep diary data manually. However, the program also offers the possibility for users to connect a wearable device to feed device-estimated sleep data into the program. In this case, device data is used to auto-fill fields in the user’s diary. If a user feels the device data did not accurately represent their sleep, they have the option to manually edit the diary data. Currently supported devices are the Jawbone UP*™* (San Francisco, CA, USA) and Fitbit® (San Francisco, CA, USA).

### Measures

Baseline and post-therapy variables are routinely assessed using a comparable set of questions. Sleep is evaluated with the Sleep Condition Indicator.^32^ The SCI is based on DSM-5 criteria for Insomnia Disorder and consists of 8 items scored on a 5-point scale (0–4), a higher score indicates better sleep. The SCI has been shown to have a robust internal consistency (*α* ≥ 0.86) and convergent validity with the Pittsburgh Sleep Quality Index (*r* = −0.734) and Insomnia Severity Index (*r* = −0.793). The SCI-8 (8-item), when used as screening measure, incorporates the item “how long have you had a problem with your sleep?” (rated as I don’t have a problem/<1 month; 1–2 months: 3–6 months: 7–12 months: >1 year). For the purposes of the present paper, and to assess the therapy effect independent of this descriptor item, we calculated the SCI-7 comprising sleep continuity and quality (3 items), daytime consequences (2 items), number of nights per week affected, and extent troubled by poor sleep.^32^ Scores are transformed into a more intuitive 0–10 SCI to facilitate interpretation. In addition, we assessed multiple related well-being components which have been shown to be improved by dCBT previously.^24–27^ First, depressive symptoms were determined using the 2-item version of the Patient Health Questionnaire (PHQ-2).^33^ This measure has been demonstrated to be a good predictor of Major Depression Disorder with a sensitivity of 83% and a specificity of 90%. Second, anxiety was assessed with a 2-item version of the Generalized Anxiety Disorder questionnaire (GAD-2) with a 65% sensitivity and 88% specificity to determine any anxiety disorder.^34^ Third, we measured perceived stress with one item of the Perceived Stress Scale (PSS):^35^ “Over the past 2 weeks, how often have you felt that you were unable to control the important things in your life?” (0 to 4 scale, “not at all” to “very often”). Of the four short-form items, this item has the strongest factor loading (*r* = 0.80) on perceived stress.^36^ Fourth, life satisfaction was assessed with the question *‘*“All things considered, how satisfied are you with your life as a whole these days?” rated on a 0 to 10 scale with 0 being extremely dissatisfied.^37^ Last, productivity at work was assessed with the item “Over the past month, to what extent has poor sleep affected your ability to get through your work?”, answer possibilities were on a 0–10 scale (“Not at all” to “Completely prevented”). This item originates from the Work Productivity and Impairment questionnaire (WPAI)^38^ and was only asked to those who were employed full-time or part-time.

To assess the level of engagement and interaction with the program, we used data stored automatically by the program. First, the number of completed daily sleep diaries is stored automatically, and represents how often a participant completed a daily diary. Participants are encouraged to complete their diaries daily during the therapy, but can create summary diaries if they have not been able to keep up with daily reporting. The number of completed diaries only includes the number of completed daily diaries. Second, we assessed interaction with program features including viewing the library, viewing the community and posting on the community. Each of these components was assessed binomially and indicated whether the person did use the respective component or not.

Finally, we collected several baseline descriptive variables including information about sex, age, shift work (yes/no), whether the user considered himself/herself overweight (yes/no), whether they used alcohol, caffeine and/or tobacco, whether they exercised on a regular basis and whether the user reported taking prescribed sleep medication (yes/no).

### Statistical analyses

Descriptive data and outcomes are presented for the full sample. Group comparisons are made for those who connected a device to automatically generate daily diary data and those who did not connect a device. Binomial data were analyzed using a Chi-square test. Continuous and ordinal data were analyzed with *t*-tests when normally distributed (paired and unpaired), and otherwise with the Mann–Whitney U-test or the Wilcoxon signed rank test. Results were stratified for baseline descriptive variables with a significant difference between users who connected a device and users who did not connect a device. IBM SPSS version 22.0 (IBM Corp., Somers, NY, USA) was used to perform all analyses.

### Data availability

Data are available on request due to privacy or other restrictions.

## Electronic supplementary material

Supplementary table 1

Supplementary table 2

## Electronic supplementary material

**Supplementary information** accompanies the paper on the *npj Digital Medicine* website (10.1038/s41746-017-0010-4).
