# Supplementary material for: Delivering digital cognitive behavioral therapy for insomnia at scale: does using a wearable device to estimate sleep influence therapy?
Source: NPJ Digit Med. 2018 Feb 19;1:3. doi: 10.1038/s41746-017-0010-4 (PMC6548338; doi:10.1038/s41746-017-0010-4)
Supplement: Supplementary file 1 — Supplementary table 1 [file 41746_2017_10_MOESM1_ESM.docx]

*Supplementary table 1 Sleep, depression, anxiety, perceived stress, overall health, life satisfaction and productivity at baseline and post-treatment for users who did not use prescribed sleep medication (n=2704) versus user who used prescribed sleep medication (n=813)*

|  | **No sleep mediation** | |  |  | **Sleep medication** | |  |  |
| --- | --- | --- | --- | --- | --- | --- | --- | --- |
|  | *Baseline* | *Post treatment* |  |  | *Baseline* | *Post treatment* |  |  |
|  | Mean ± SD/ Median (IQR) | Mean ± SD/ Median (IQR) | Test-statistic | p | Mean ± SD/ Median (IQR) | Mean ± SD/ Median (IQR) | Test-statistic | p |
| Sleep (SCI-7) | 4.58 ± 1.95 | 7.37 ± 1.96 | t(2673)=-71.83 ^1^ | <0.001 | 3.19 ± 1.45 | 6.45 ± 2.12 | t(802)=-42.50 ^1^ | <0.001 |
| Depression (PHQ-2) | 1 (0-3) | 0 (0-2) | Z=-23.43 ^2^ | <0.001 | 2 (1-3) | 1 (0-2) | Z=-13.00 ^2^ | <0.001 |
| Anxiety (GAD-2) | 2 (0-3) | 1 (0-2) | Z=-28.84 ^2^ | <0.001 | 2 (1-4) | 1 (0-2) | Z=-15.62 ^2^ | <0.001 |
| Perceived stress (PSS) | 2 (1-2) | 1 (0-2) | Z=-25.00 ^2^ | <0.001 | 2 (1-3) | 1 (0-2) | Z=-13.73 ^2^ | <0.001 |
| Life satisfaction | 7 (6-8) | 7 (6-8) | Z=-17.00 ^3^ | <0.001 | 7 (5-8) | 7 (6-8) | Z=-8.88 ^3^ | <0.001 |
| Work productivity | 2 (1-3) | 1 (0-2) | Z=-21.81^2^ | <0.001 | 3 (2-5) | 2 (0-3) | Z=-13.19 ^2^ | <0.001 |

SD: Standard deviation; IQR: interquartile range; SCI-7: Sleep Condition Indicator 7 items; PHQ-2: Patient Health Questionnaire 2 items; GAD-2: Generalize Anxiety Disorder 2 items.

^1^ Paired T-test

^2^ Wilcoxon signed rank test, Z-value based on negative ranks

^3^ Wilcoxon signed rank test, Z-value based on positive ranks
